# Primary pulmonary T-cell lymphoma with lung adenocarcinoma: a case report

**DOI:** 10.1186/s13256-019-2091-y

**Published:** 2019-06-30

**Authors:** Yutaka Takahara, Takashi Sakuma, Kazuaki Nishiki, Keisuke Nakase, Masafumi Nojiri, Yoshimichi Ueda, Shiro Mizuno

**Affiliations:** 1 Department of Respiratory Medicine, Kanazawa Medical University, 1-1 Daigaku, Uchinada-machi, Kahoku-gun, Ishikawa 920-0293 Japan; 2 Department of Pathology, Kanazawa Medical University, 1-1 Daigaku, Uchinada-machi, Kahoku-gun, Ishikawa 920-0293 Japan

**Keywords:** Primary pulmonary T-cell lymphoma, Lung adenocarcinoma, Double primary cancer

## Abstract

**Background:**

Primary pulmonary T-cell lymphoma is an extremely rare disease that is characterized by neoplastic proliferation of T-cell lymphocytes in the lung.

**Case presentation:**

An 88-year-old Japanese woman was admitted to our hospital because of dyspnea. She was treated with antibiotics for chest x-ray findings of consolidation in the upper and middle lobes of the right lung. However, her condition worsened and progressed to respiratory failure, which led to death 14 days after admission. Autopsy of the lungs revealed both T-cell lymphoma and adenocarcinoma.

**Conclusions:**

Our patient had a rare case of primary pulmonary T-cell lymphoma with primary lung adenocarcinoma. Further evidence and accumulation of case reports are required to clarify the pathophysiology of primary pulmonary T-cell lymphoma.

## Background

Primary pulmonary lymphoma is a rare neoplasm, with a reported frequency of less than 0.5% of all primary lung tumors and less than 1% of all lymphomas [1]. Moreover, about 95% of primary pulmonary lymphoma cases are of B-cell origin and are commonly derived from bronchial mucosa-associated lymphoid tissue and diffuse large B-cell lymphoma [2, 3]. However, little is known about primary pulmonary lymphoma of T-cell origin. In addition, most lung cancers that are associated with malignant lymphoma are secondary to chemotherapy and/or radiation therapy. Double primary lung cancer, which is unrelated to these treatments, is extremely rare. This is a rare and important report of primary pulmonary T-cell lymphoma with lung adenocarcinoma pathologically confirmed by autopsy.

## Case presentation

**Fig. 1:**
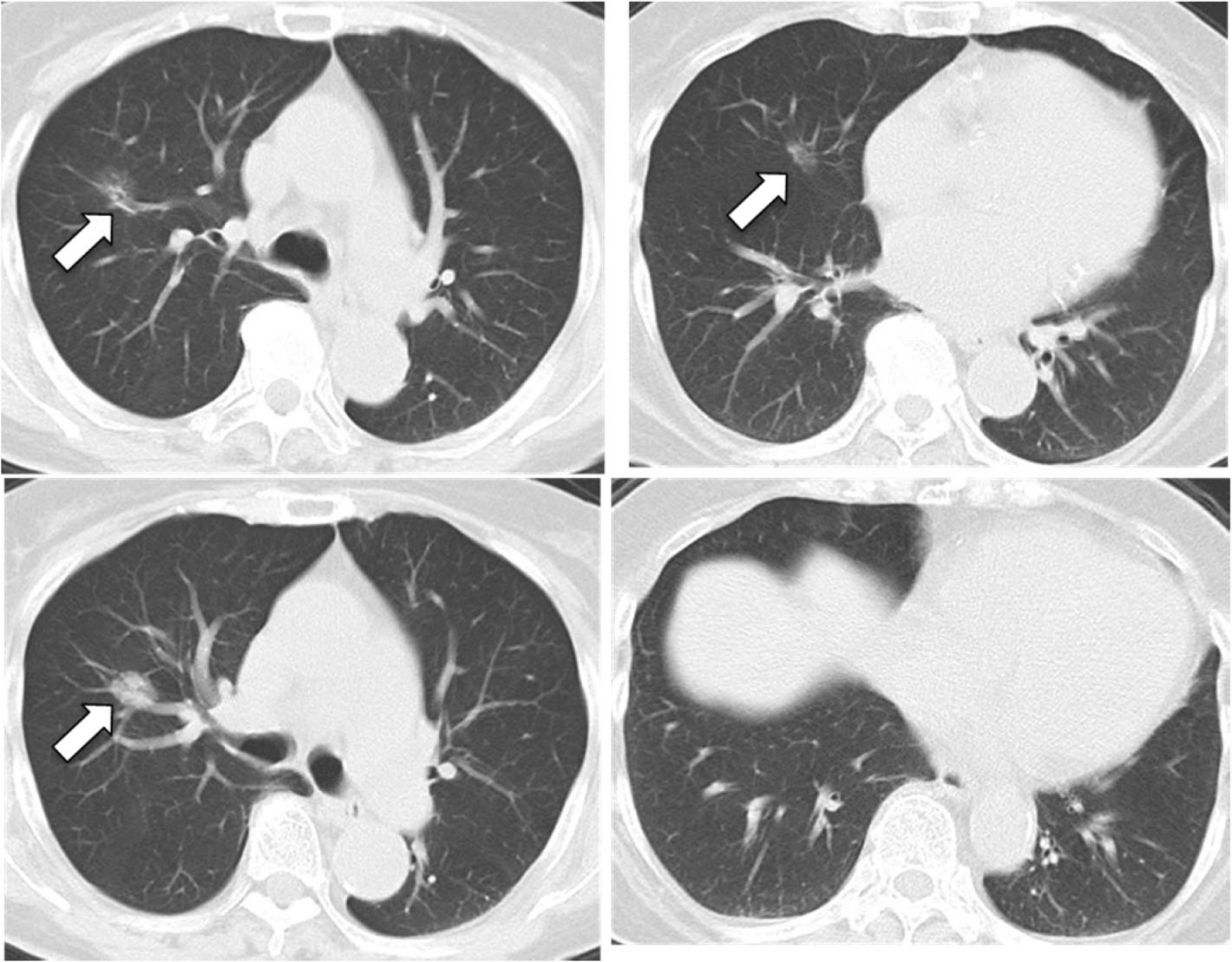
Chest computed tomographic images obtained at the first visit. Chest computed tomography upon initial examination shows a mixed ground-glass opacity nodule (*arrows*) in the upper and middle lobes of the right lung

An 88-year-old Japanese woman was noted to have an abnormal chest shadow, which was characterized on the basis of computed tomography (CT) as a mixed ground-glass opacity nodule in the upper and middle lobes of the right lung (Fig. 1). She had no symptoms. Because of her age, the patient and her family did not desire further examination. A tentative diagnosis of bronchioloalveolar carcinoma was made. Follow-up chest CT every 3 to 6 months for 15 months showed stability of the pulmonary nodules. Fifteen months after the first visit, she was referred to our hospital from a nearby clinic because of dyspnea, mild cough, and chest x-ray findings of consolidation in the right lung.

She had a medical history of hypertension, for which she was receiving oral azilsartan 40 mg once daily and amlodipine 5 mg two times daily. Her social and family history was unremarkable, and she had no smoking history. Her environmental history revealed no abnormalities. She was a housewife.

Her physical examination upon admission revealed blood pressure of 140/70 mmHg, pulse rate of 120 beats per minute, temperature of 37.9 °C, and percutaneous oxygen saturation of 91% on room air. Coarse crackles at the right lung base were noted, but she had no palpable superficial lymph nodes. The result of her cardiovascular examination was normal, and her abdominal examination was unremarkable with no hepatosplenomegaly. A neurological examination revealed no abnormalities, and no skin lesions were appreciated.

**Fig. 2:**
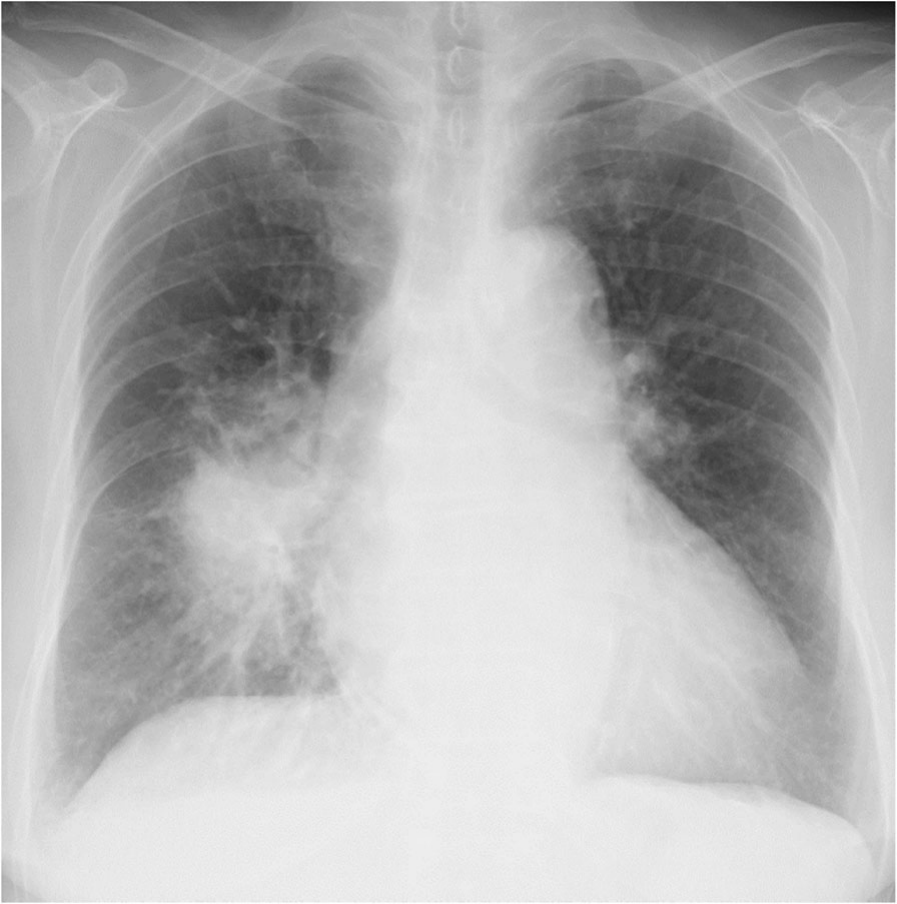
Chest x-ray obtained upon admission. Chest radiography reveals areas of consolidation, mainly in the right lower lung

**Fig. 3:**
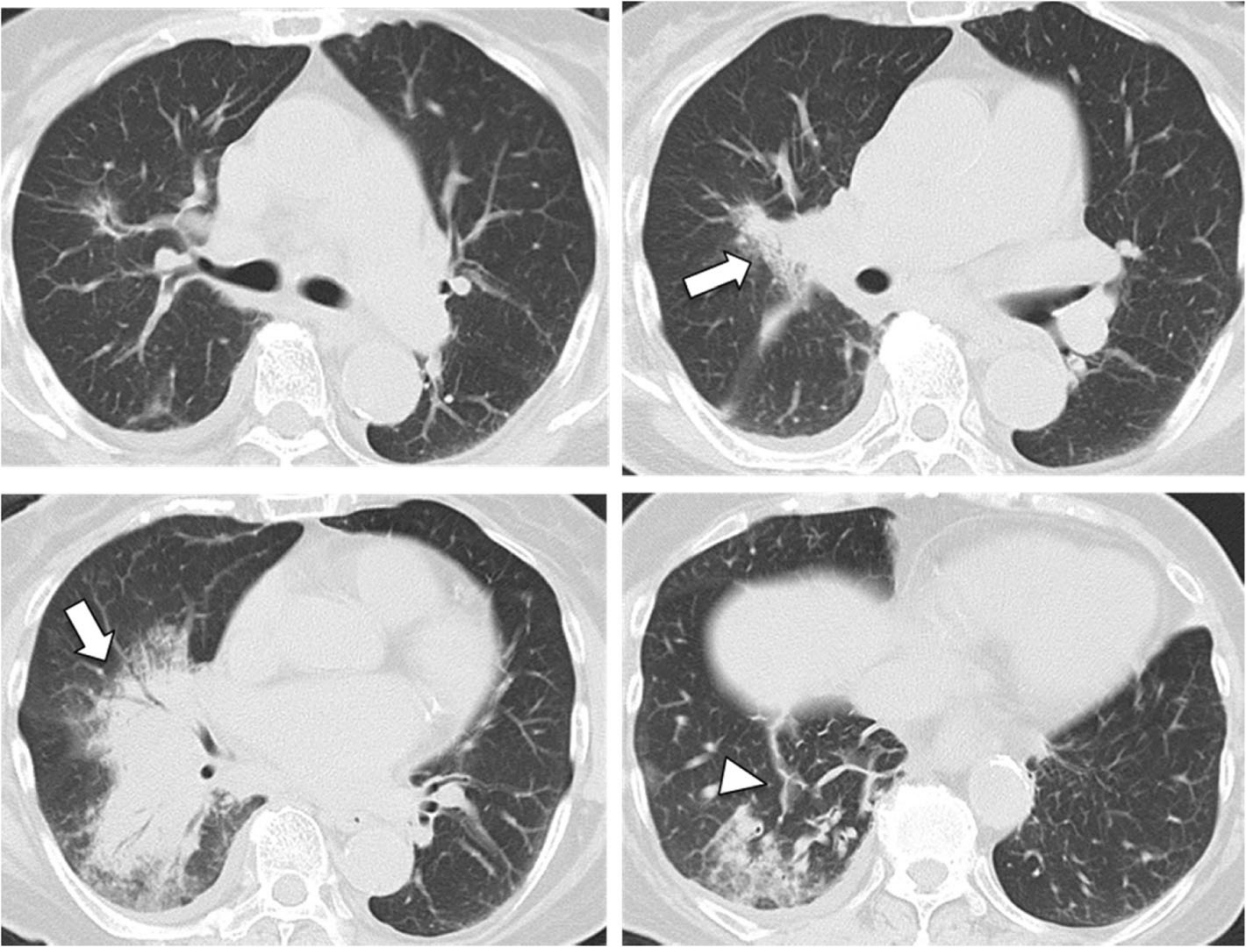
Chest computed tomography on admission. Chest computed tomographic scan reveals areas of consolidation with ground-glass opacities (*arrows*) and interlobular septal thickening in the right middle and lower lobes (*arrowheads*)

Laboratory findings showed a normal white blood cell count (6200/μl) and elevated levels of serum C-reactive protein (14.25 mg/dl) and carcinoembryonic antigen (6.8 ng/ml). The laboratory findings showed that serum aspartate aminotransferase was 76 U/L, serum alanine aminotransferase was 65 U/L, and lactate dehydrogenase was 364 U/L. The serum levels of electrolytes, creatinine, and blood urea nitrogen were normal. Antineutrophil cytoplasmic antibody was negative. Sputum culture results were all negative for a causative organism. A chest x-ray showed areas of consolidation, mainly in the right lower lung (Fig. 2). Chest CT images showed areas of consolidation in the right middle and lower lobes with ground-glass opacities and interlobular septal thickening (Fig. 3).

**Fig. 4:**
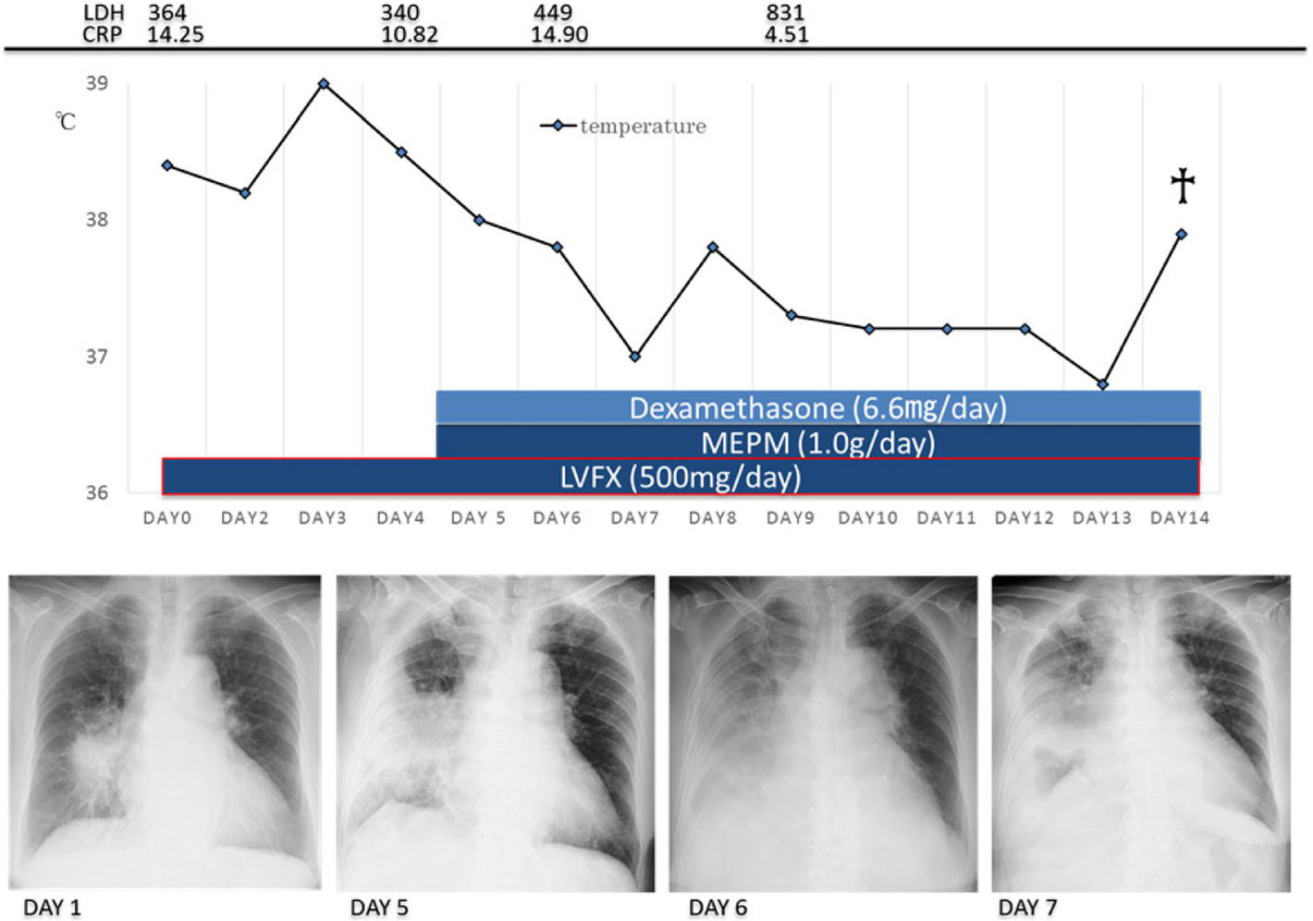
The patient’s clinical course, including laboratory data, radiologic findings, and treatment. *LVFX* Levofloxacin, *MEPM* Meropenem

Upon admission, she was treated with intravenous levofloxacin (500 mg/day). However, her symptoms and chest radiologic findings worsened; therefore, intravenous meropenem (1 g/day) was added. In addition, dexamethasone (6.6 mg/day) was started because of the possibility of carcinomatous lymphangitis. The patient and her family did not wish for her to receive aggressive medical treatment, so we did not perform bronchoscopy. Corticosteroid therapy temporarily ameliorated the patient’s fever and dyspnea, but she rapidly developed respiratory failure and died 14 days after disease onset. Her clinical course is shown in Fig. 4.

**Fig. 5:**
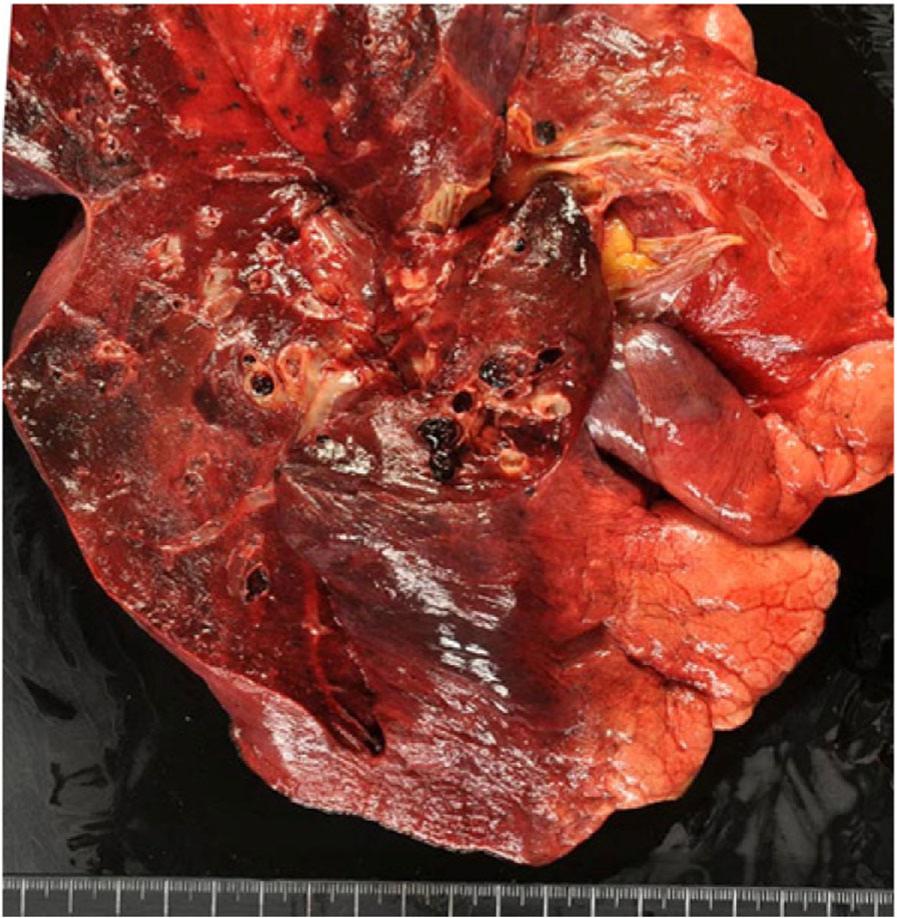
Macroscopic findings of the lung. Macroscopically, pulmonary hemorrhage and pulmonary artery thrombus are observed in the bilateral lower lobes

**Fig. 6:**
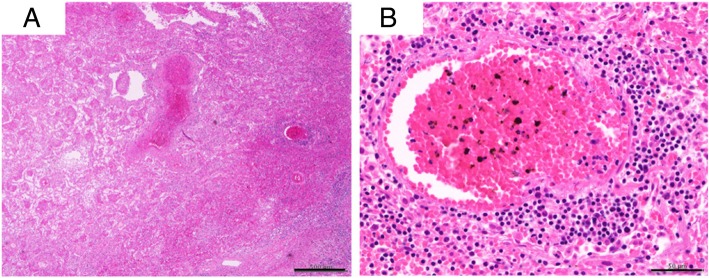
Histologic examination of the lung shows perivascular infiltration of small to medium-sized lymphoid cells with nuclear atypia. **a** Hematoxylin and eosin (H&E) staining, scale bar = 500 μm. **b** H&E staining, scale bar = 50 μm]

**Fig. 7:**
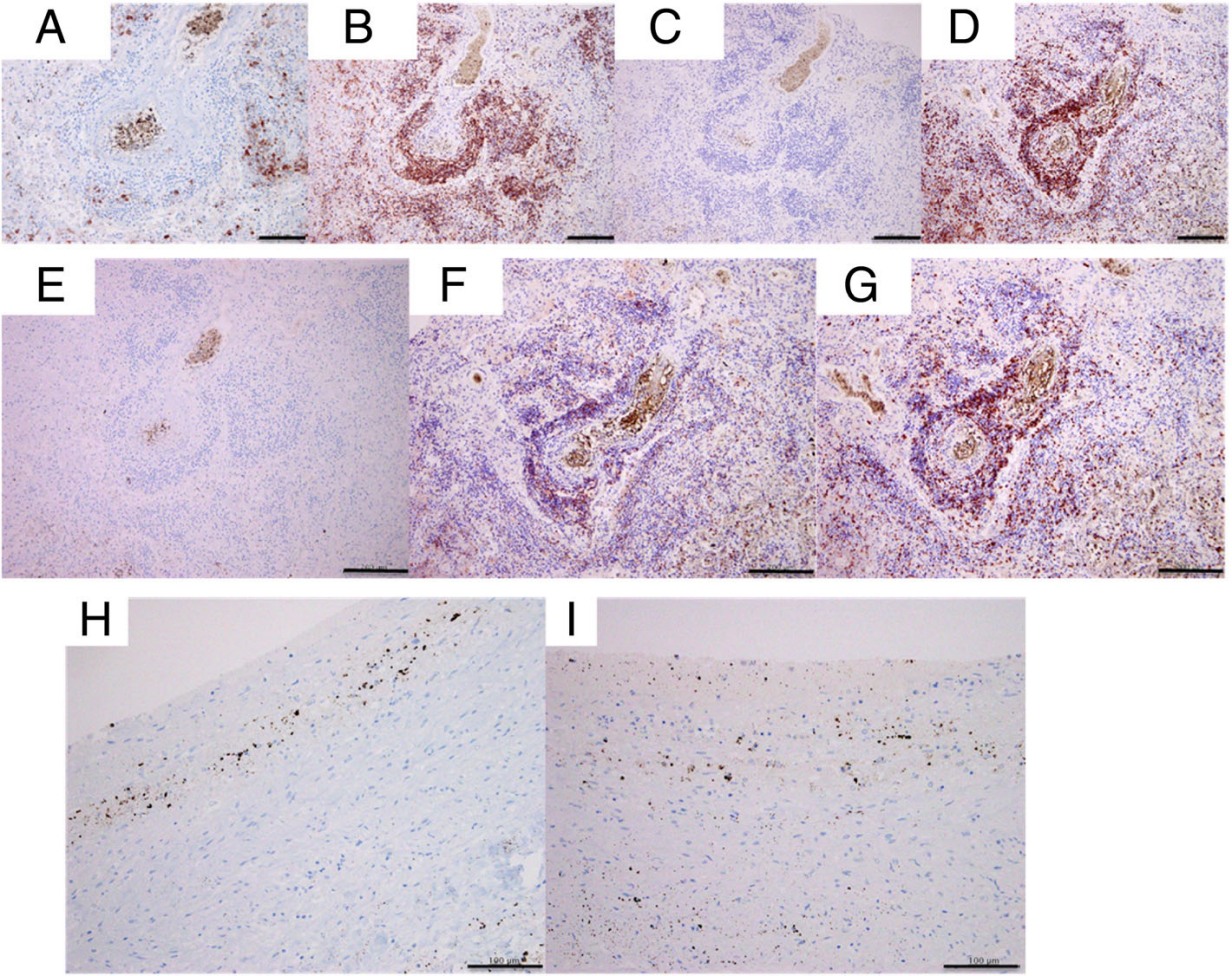
Immunohistochemical staining of the lungs shows that the tumor cells are positive for CD3, CD7, and CD4 but negative for CD20, CD3, CD5, CD56, granzyme B, and perforin. **a** CD20 staining, scale bar = 100 μm. **b** CD3 staining, scale bar = 200 μm. **c** CD56 staining, scale bar = 200 μm. **d** CD7 staining, scale bar = 200 μm. **e** CD5 staining, scale bar = 200 μm. **f** CD4 staining, scale bar = 200 μm. **g** CD8 staining, scale bar = 200 μm. **h** Granzyme B staining, scale bar = 100 μm. **i** CD perforin staining, scale bar = 100 μm

After consent from her family was secured, an autopsy was performed, which revealed hemorrhagic infarction of the bilateral lower lobes of the lungs due to pulmonary arterial thrombosis (Fig. 5). Histologic examination of the lungs showed bilateral diffuse infiltration of atypical lymphocytes in the alveolar septum and pulmonary arteries, with thrombus formation (Fig. 6). The infiltration of atypical lymphocytes was found in the bilateral lower lobe mainly, bilateral upper leaves, and middle right lobe. Immunohistochemical (IHC) analysis showed that the atypical lymphocytes were positive for CD3, CD4, CD8, and CD7 and negative for CD20, CD5, CD56, granzyme B, and perforin (Fig. 7). Epstein-Barr virus-encoded small RNAs were negative on the basis of *in situ* hybridization analysis. T-cell receptor (TCR)-β and TCR-γ gene rearrangement was not detected by polymerase chain reaction (PCR) analysis.

**Fig. 8:**
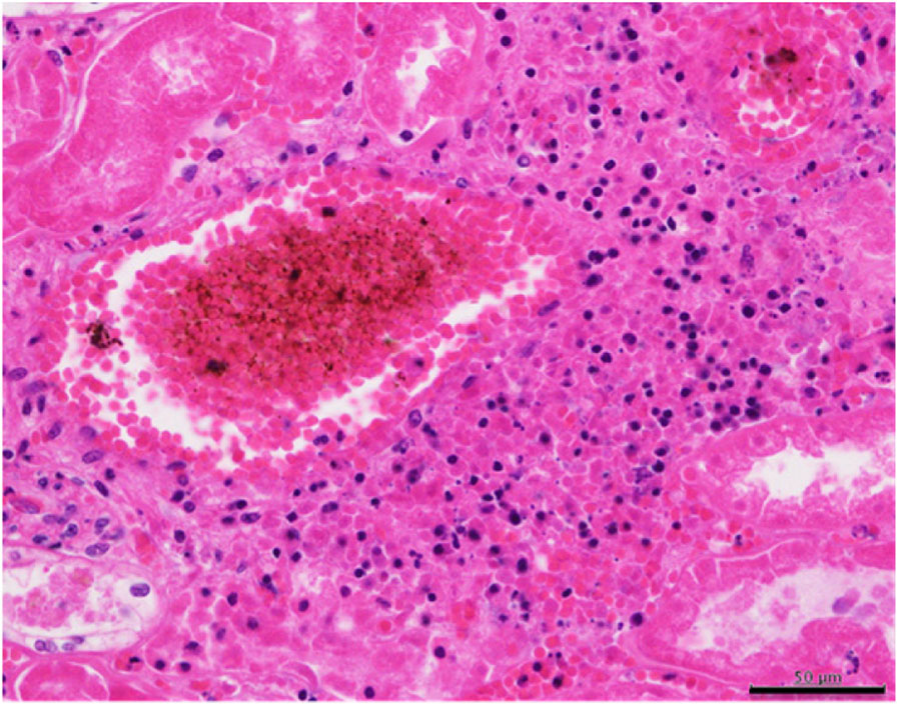
Histologic examination of the kidney. Thrombus formation and atypical lymphocyte infiltration are observed in the bilateral renal arteries, with hemorrhagic infarction in both kidneys. H&E staining, scale bar = 500 μm

**Fig. 9:**
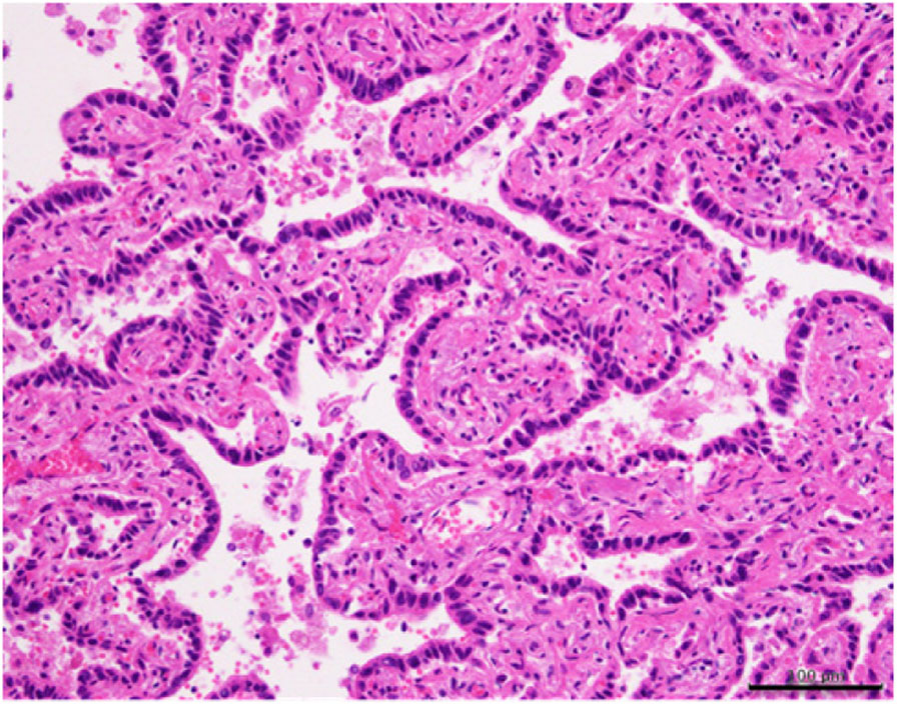
Histologic examination of the lung. Well-differentiated adenocarcinoma with a bronchioloalveolar carcinoma growth pattern is observed in the right upper lobe S^3^. H&E staining, scale bar = 100 μm

Based on these findings, a diagnosis of peripheral T-cell lymphoma was made. Further review showed atypical lymphocyte infiltration with resulting thrombus formation in the bilateral renal arteries and hemorrhagic infarction of both kidneys (Fig. 8). The atypical lymphocyte infiltration was not observed in the other organs, except in the lungs and kidneys. Moreover, well-differentiated adenocarcinoma with a bronchioloalveolar carcinoma growth pattern was observed in the right upper lobe S^3^ of the lung, but there were no lymphatic or hematogenous metastases (Fig. 9).

## Discussion

Our patient had coincidental primary pulmonary T-cell lymphoma and primary lung adenocarcinoma. Little has been reported about primary pulmonary T-cell lymphoma. In addition, to the best of our knowledge, only one case of concomitant primary pulmonary T-cell lymphoma and lung cancer has been reported to date. It is extremely rare to observe a case of such synchronous double cancers.

To the best of our knowledge, only 16 primary pulmonary T-cell lymphoma cases, including our patient’s, have been reported since 1991 [4]. Yang *et al.* reviewed 15 cases of primary pulmonary T-cell lymphoma and reported nonspecific chest radiologic features, including multiple nodules (8 of 15), masses (2 of 15), consolidations (2 of 15), pleural effusion (1 of 15), patchy infiltration (1 of 15), ground-glass opacities (1 of 15), reticular shadows (1 of 15), and emphysema (1 of 15). The most common radiologic finding was the presence of multiple nodules, which occurred in 53.3% of all patients.

In our patient, the chest CT images showed ground-glass opacities, consolidation, and interlobular septal thickening, but not multiple nodules. Moreover, the dense consolidation was newly appeared in the right middle and lower lobes upon admission. In the examination by autopsy, adenocarcinoma was observed only in right upper lobe S^3^. Apart from that, only infiltration of atypical lymphocytes and hemorrhagic necrosis were observed. Therefore, the consolidation suggested the possibility of disease progression of the old lesion. However, histologic examination of ground-glass shades of the right middle lobe and the right lower lobe was not done at an early stage. Also, because the natural history of primary pulmonary T-cell lymphoma is unknown, the onset time in our patient’s case is unknown.

In hindsight, the interlobular septal thickening visualized by chest CT could have been due to infiltration of atypical lymphocytes in the lymphatic vessels. Moreover, it was possible that the growth pattern of the atypical lymphocytes in our patient was infiltration through the lymphatic vessels, in addition to direct invasion and hematogenous metastasis. On the basis of our patient’s case, we speculate that the nonspecific radiologic features of primary pulmonary T-cell lymphoma could be caused by the varied growth pattern. Previous studies showed that the pathologic features of this primary pulmonary T-cell lymphoma comprise cells of intermediate to large size, with moderate pleomorphism and extensive necrosis in the angiocentric lesions. The pathologic findings in this case suggested that the necrosis of tumor cells and lung tissue were secondary to tumor development and thrombus formation.

In our patient, we found the coexistence of adenocarcinoma in the right upper lobe S^3^ of the lung and T-cell lymphoma. In a report by Kondo *et al.* [5] on 731 cases of resected lung cancer, the frequency of multiple primary cancers, particularly with coexisting malignant lymphoma, was rare at 0.8%. In addition, only one case of T-cell lymphoma and primary lung cancer overlap has been reported [6]. In that report of a rare case by Kawashima *et al*., squamous cell carcinoma was demonstrated to have coexisted with T-cell lymphoma in the lungs, but there was no explanation on the association between the two diseases. Chronic inflammatory conditions such as Epstein-Barr virus infection and immunosuppression were thought to be the cause of malignant lymphoma [7]. However, most of the pathogenic mechanisms of primary pulmonary T-cell lymphoma remain uncertain. Because of the rarity of this overlap, we supposed that there was no association between lung cancer and primary pulmonary T-cell lymphoma.

The clinical course of T-cell lymphoma of the lung is usually aggressive, and the respiratory failure resulting from rapid tumor progression leads to death. The standard treatment of the primary pulmonary T-cell lymphoma has not been established, owing to the scarcity of evidence [8]. Accumulation of cases is required in order to understand the pathophysiology of this disease.

## Conclusions

We report the pathological correlation between the autopsy findings and nonspecific chest radiologic features on CT in a patient with primary pulmonary T-cell lymphoma. Although this is a very rare case, physicians should include primary pulmonary T-cell lymphoma in the differential diagnosis of patients with dyspnea, fever, and chest CT findings of ground-glass opacities, consolidation, and interlobular septal thickening.

## Disclosures

This case was presented at a case conference during the 72nd annual meeting of the Japan Lung Cancer Society on July 8, 2017. The authors state that they have no conflict of interest with regard to this report.
